# Comparison of cysteine content in whole proteomes across the three domains of life

**DOI:** 10.1371/journal.pone.0294268

**Published:** 2023-11-13

**Authors:** Adriana Castillo-Villanueva, Horacio Reyes-Vivas, Jesús Oria-Hernández

**Affiliations:** Laboratorio de Bioquímica-Genética, Instituto Nacional de Pediatría, Secretaría de Salud Ciudad de México, Ciudad de México, México; Universidad Autónoma Agraria Antonio Narro, MEXICO

## Abstract

An empirical observation suggests that *Giardia lamblia* proteins have larger cysteine content than their counterparts in other organisms. As this parasite lacks conventional antioxidant stress systems, it is generally accepted that high cysteine content helps *G*. *lamblia* cope with oxygen toxicity, a strategy apparently shared by other organisms. Here, we question whether the high cysteine content in some organisms is genuine or just a simple assumption based on singular observations. To this end, we analyzed the cysteine content in 78 proteomes of organisms spanning the three domains of life. The results indicate that the cysteine content in eukaryota is approximately double that in archaea and bacteria, with *G*. *lamblia* among the highest. Atypical cysteine contents were found in a few organisms correlating with specific environmental conditions, supporting the evolutionary amino acid-level selection of amino acid composition.

## Introduction

Cysteine is a unique amino acid within the protein chemistry; it is the only one that contains a sulfhydryl group in its side chain. In addition to the classical thiol-disulfide redox equilibria, cysteine undergoes a broad range of redox modifications that regulate protein function at diverse levels, including ligand binding and catalysis, protein stability and protein‒protein interactions [1]. In light of these data, it is interesting to note that cysteine residues have a lower frequency than expected; considering 61 coding codons and the presence of only two for cysteine (UGU and UGC), the expected frequency is 3.28%, a higher value than that found in nature (1.7% [2]). It seems then that cysteine has been selected against in the course of evolution.

While working with recombinant proteins from the parasite *Giardia lamblia* (*syn*. *G*. *intestinalis*, *G*. *duodenalis*) [3–5], we empirically observed that they appeared to show a higher cysteine content in comparison with their counterparts from other genera. From a comparative study of the cysteine content of glycolytic enzymes in a small group of organisms, this observation was repeated.

Cysteine is an essential amino acid *for G*. *lamblia*; it has been reported that high cysteine concentrations are needed in the culture media for the growth of trophozoites (the vegetative form of *Giardia*) [6], with cysteine being the major thiol group present in the cell [7]. *G*. *lamblia* does not synthesize cysteine; therefore, it depends on its assimilation from the media. Most of the acquired cysteine is incorporated into proteins, especially into one family of cysteine-rich proteins (variant-specific proteins, VSPs) [6]. It is generally accepted that a high cysteine content helps *G*. *lamblia* cope with oxygen toxicity [6, 8, 9], and it has been suggested that VSPs may play a role in the protection of the trophozoite from oxygen toxicity [6]. It is important to highlight that *G*. *lamblia* is a strictly microaerophilic parasite that lacks conventional antioxidant stress systems such as superoxide dismutase, catalase, peroxidase, glutathione peroxidase, and glutathione reductase, therefore requiring alternative defenses [10]. It is interesting to note that this same strategy (proteins with a high cysteine content as a protective system against oxidative stress) has been proposed in other parasites, such as *Entamoeba histolytica* and *Tritrichomonas fetus* [8], pathogens, such as *Phytophthora cactorum* [11], and plants, such as *Arabidopsis thaliana* [12].

In this work, we question whether the high cysteine content in some organisms is genuine or just a simple assumption based on singular observations of isolated proteins. To this end, we performed a proteome-wide comparison of cysteine content in a variety of organisms spanning all 3 domains of life and statistically analyzed them. The results indicate that, overall, the cysteine content is higher in eukaryotes than in archaea and bacteria, with *G*. *lamblia* among the highest. Only a few organisms show higher or lower outliers within each of their groups, indicating that claims of atypical cysteine content in many organisms may be biased by incidental observations. Atypical cysteine contents can be related to specific environmental conditions, supporting the evolutionary amino acid-level selection of amino acid composition.

## Methods

### Database

The sets of proteomes were retrieved from the Reference Proteome web page, version RELEASE 2022_02, hosted on the EMBL’s European Bioinformatics Institute site (https://www.ebi.ac.uk/reference_proteomes/). The Reference Proteomes from The Quest for Orthologs Group include 78 proteomes, including members of the 3 domains of life (7 Archaea, 23 Bacteria and 48 Eukaryota). This dataset comprises curated, complete, standardized and nonredundant proteomes intended to sample a broad taxonomic space [13]. It is important to highlight that homogeneous, highly reliable databases are essential to guarantee the quality control of the input data and therefore of the obtained results. The full list of organisms and their proteome identifiers and abbreviations is provided as S1 Datasheet.

### Data analysis

The amino acid composition for each proteome was performed with the code Perl *aacomposition*.*pl* [14], generously provided by Dr. Pablo Mier from the Computational Biology and Data Mining group at the Johannes-Gutenberg University of Mainz, Germany. From the complete set of amino acid composition data for all proteomes, only cysteine data were extracted for analysis in this work. Statistical analysis and data plotting were performed using Prism and Origin software. The percentage of cysteine content for each organism and the numerical values of the statistical analysis for each domain are provided as S2 Datasheet.

Individual aminoacyl sequences from the enzymes of the glycolytic pathway were searched in the OrthoDB v11 database [15] and retrieved from UniProt [16]; cysteine content for each sequence was calculated with ProtParam [17].

## Results

### Cysteine content differs between domains of life

**Fig 1:**
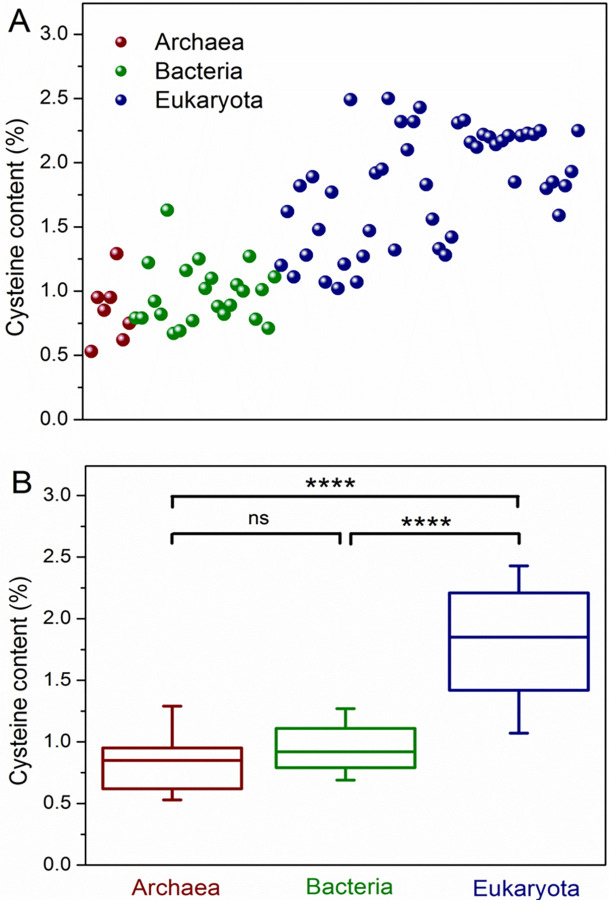
Distribution of cysteine content according to each domain of life. A) Each point represents an organism in relation to the percentage of cysteine in its entire proteome. B) Kruskal‒Wallis test with Dunn’s multiple comparisons posttest. Boxes represent the median and the 25th and 75th percentiles of the data, whereas bars indicate the 5th and 95th percentiles; ns: not significant; ****: p < 0.0001.

Fig 1A shows a clear tendency for an increase in cysteine content when going from archaea and bacteria to eukaryota. To demonstrate whether these differences were significant, we performed a statistical comparison between cysteine content for the 3 life domains. Since the data did not follow a normal distribution in any case, the median and percentiles of the data were used for this analysis (Fig 1B). The results showed that there was no significant difference between the groups of archaea and bacteria, but in both comparisons, eukaryota *vs*. bacteria or eukaryota *vs*. archaea, the statistical significance was high (p < 0.0001). The results indicate that the cysteine content in eukaryota is the highest among the 3 domains of life.

### Cysteine content is independent of proteome size

It is possibly that differences in proteome dimensions could account for the differences in cysteine occurrence; therefore, we analyze the correlation between proteome size and cysteine content (S1 Fig). The results showed that in archaea and bacteria there was no such correlation. For archaea (S1 Fig panel A), the Pearson correlation coefficient (r) was 0.016 with p = 0.972, whereas for bacteria (S1 Fig, panel B), the results were r = 0.017 and p = 0.937. In eukaryota the values were r = 0.365 with p = 0.017, indicating a low correlation; however, the dispersion is notable as can be observed (S1 Fig, panel C). Overall, the data indicates that the cysteine content is not related to proteome size.

### Few organisms show atypical cysteine content values

**Fig 2:**
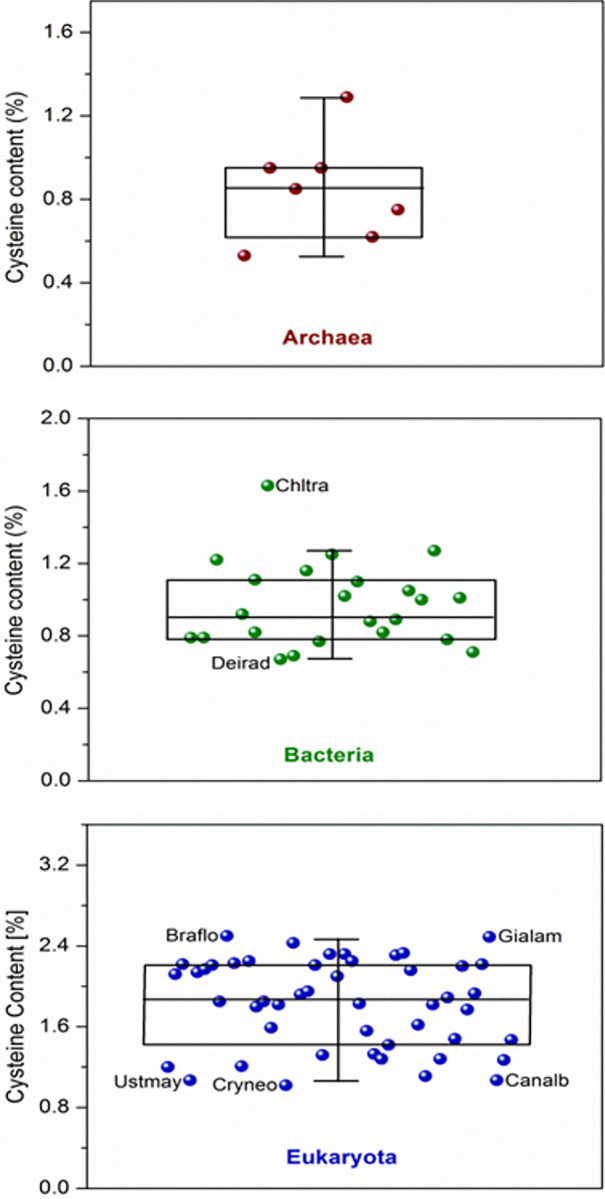
Cysteine content for each organism within its life domain. For all panels, each point represents an organism within the statistical analysis of its group. Outliers are indicated by an abbreviation consisting of the first 3 letters of the genus and species (Chltra: *Chlamydia trachomatis* in panel B, for example), in consonance with the abbreviations used in the S2 Datasheet.

As a whole, only a few organisms displayed atypical cysteine content values; out of 78 proteomes, 3 showed high outliers and 4 low outliers, representing only 9% of the total. We defined outliers as those below or above the 5% and 95% percentiles, respectively, within their own groups. For the archaea domain, no outliers were found (Fig 2A and S2 Datasheet). In contrast, in the bacteria domain, *Chlamydia trachomatis* showed a cysteine content of 1.63%, a significantly higher value than that of 1.27% of the 95th percentile of the group. On the other hand, *Deinococcus radiodurans* presents a cysteine content below the 5th percentile of the group, 0.667% *vs* 0.67%, respectively (Fig 2B and S2 Datasheet). Finally, in the eukaryota domain (95% and 5% percentiles = 2.46% and 1.07%, respectively), 2 upper outliers, *Giardia lamblia* (2.49%) and *Branchiostoma floridae* (2.5%), were found, while 3 lower outliers were observed, *Cryptococcus neoformans* (1.02%), *Candida albicans* (1.067%), and *Ustilago maydis* (1.068%) (Fig 2C and S2 Datasheet). The results confirm our empirical observation that *G*. *lamblia* has one of the highest cysteine contents among organisms from all domains of life. In addition, other organisms with atypical (lower or higher) cysteine content values were uncovered. The possible significance of these results is discussed below.

### Cysteine content consistently differs in individual proteins

Cysteine content was analyzed in the individual enzymes of the glycolytic pathway for ten organisms, one with high cysteine content (*G*. *lamblia*), with high cysteine content (*G*. *lamblia*) and another with low cysteine content (*C*. *albicans*); additionally, eight model organisms, from amoeba to human, were used for comparison. (*Dictyostelium discoideum*, *Caenorhabditis elegans*, *Arabidopsis thaliana*, *Drosophila melanogaster*, *Xenopus tropicalis*, *Danio rerio*, *Mus musculus* and *Homo sapiens*); glycolysis was selected because it is a highly conserved pathway among living organisms. The results show that *G*. *lamblia* proteins consistently, although not in all cases, have higher cysteine content than their counterparts; accordingly, *C*. *albicans* enzymes show a lower cysteine content. When analyzed as a whole, the average cysteine content of the enzymes from *G*. *lamblia* is the highest, whereas in *C*. *albicans* is the lowest (Table 1). The individual protein data are in good agreement with the proteomic results.

**Table 1:** Cysteine content of individual enzymes from the glycolytic pathway.

Enzyme/Organism	*G*. *lamblia*	*C*. *albicans*	*D*. *discoideum*	*C*. *elegans*	*A*. *thaliana*	*D*. *melanogaster*	*X*. *tropicalis*	*D*. *rerio*	*M*. *musculus*	*H*. *sapiens*
Hexokinase/Glucokinase	2.9	1.6	3.5	2.4	1.4	3.1	2.4	2.6	2.4	2.3
Glucose-6-phosphate isomerase	2.9	0.0	0.5	0.3	0.7	0.9	0.7	0.4	0.7	0.8
Phosphofructokinase	2.9	1.2	1.4	2.2	2.2	2.7	2	2	2.6	2.1
Fructose bisphosphate aldolase	1.6	0.8	1.4	1.4	1.0	2.5	2.6	2.5	2.4	2.5
Triosephosphate isomerase	2.0	2.0	1.6	0.8	1.3	2.0	2.0	2.0	2.4	2.0
Glyceraldehyde-3-phosphate dehydrogenase	3.9	0.6	1.2	0.9	0.6	0.9	2.1	1.2	1.4	0.9
Phosphoglycerate kinase	2.9	0.5	1.2	0.5	0.4	1.2	1.7	1.9	1.7	1.7
Phosphoglycerate mutase	2.2	1.4	0.7	1.9	1.8	2.7	1.2	1.2	1.2	1.2
Phosphopyruvate hydratase (enolase)	2.5	0.0	0.9	0.7	1.5	0.9	1.4	1.4	1.6	1.4
Pyruvate kinase	3.3	1.2	1.8	2.4	2.4	2.0	1.5	1.7	1.7	1.6
Average	2.71	0.93	1.42	1.35	1.33	1.89	1.76	1.69	1.81	1.65

## Discussion

The cysteine content is not homogeneous among the 3 domains of life; the content in eukaryota doubles the values of bacteria and archaea (median values 1.87%, 0.91% and 0.85%, respectively). For eukaryota, the value is consistent with the value found in classical reference tables (1.7%) [2] but is not representative of the values from bacteria and archaea. The result indicates that at least for this amino acid, reference cysteine content values cannot be generalized if not individualized by life domain.

Although is true that higher cysteine content in proteins for eukaryota has been claimed in previous works [14, 18], 3 points need to be emphasized: 1) In the first work [14], the number of analyzed proteomes was considerably less (38 proteomes: 12 archaea, 9 bacteria and 17 eukaryota), and no analytical test was shown in order to support the conclusion that cysteine content is higher in eukaryota. 2) In the second paper [18], representative samples of proteins from 16 organisms were analyzed (3 archaea, 4 bacteria and 9 eukaryota); representative samples consist of protein sequence files for each organism where the number of protein sequences ranges from 58 in *Rhodobacter sphaeroides* to 3151 in *Escherichia coli* (that is, only a small fraction of the proteomes was sampled). 3) In this work, a curated database of 78 whole proteomes of species covering a wide phylogenetic space was statistically analyzed, making the obtained results more reliable and supporting the conclusion raised in these previous works. A higher occurrence of cysteine has been related to a higher complexity of the organisms [18], but as shown here, cysteine content is independent of the proteome size. Furthermore, the highest cysteine content in eukaryotes was found in *G*. *lamblia* and *B*. *floridae* and not in more complex organisms, as would be expected if the proposal were true.

While it is true that the number of organisms in the archaea group is small in this work, each member analyzed falls within 7 different branches of a recently described complete phylogenetic tree [19], indicating that the findings described here for this group are significant. Once a larger number of curated archaeal proteomes becomes available, these inferences could be supported. As a stringent criterion was selected to assign the outliers (cysteine percentage values above or below the 95% and 5% percentiles), these represent extreme cases of selective amino acid composition worth examining individually, as described below.

In the bacterial group, 1 upper outlier (*C*. *trachomatis*) and 1 lower outlier (*D*. *radiodurans*) were found. *C*. *trachomatis* is an obligate aerobic intracellular parasite that is the leading cause of sexually transmitted diseases and infectious blindness worldwide. The life cycle of this parasite consists of two stages: the dispersal-infective form known as the elementary body and the intracellular-replicative form named the reticulate body. The elementary body is enveloped by a protein shell called the chlamydial outer membrane complex (COMC); interestingly, this COMC is mainly composed of cysteine-rich proteins (OmcA, OmcB, MOMP and PMPs, mostly). It has been reported that COMC is stabilized by an extensive network of disulfide bonds between MOMP monomers, MOMPs and MOMP isoforms, MOMPs and cysteine-rich proteins, and MOMPs and the elementary body surface, helping to maintain the integrity of the chlamydial infectious particle [20]. Thus, the high cysteine content of *C*. *trachomatis* may be related to this particular morphological feature of the parasite.

In contrast, the lowest outlier was that of *D*. *radiodurans*, a polyextremophile known for its extreme resistance to hazardous conditions such as cold, desiccation and oxidative stress but particularly to high doses of ionizing radiation (hence its name). A synergistic combination of efficient systems for DNA repair and protection against oxidative stress seems to explain the resilience of this bacterium [21]. While DNA damage was traditionally the focus of sensitivity to ionizing radiation, it is now widely recognized that a combination of strategies to combat oxidative stress is crucial for desiccation and radiation resistance of *D*. *radiodurans*, which induce oxidative damage, especially at the protein level [21, 22]. Consistent with our work, experimental evidence comparing *Escherichia coli* and *D*. *radiodurans* proteomes by shotgun redox proteomics indicates a significant decrease in the prevalence of ROS-sensitive amino acids, especially sulfur-containing amino acids (50% less for cysteine), in the latter bacterium [22].

Among eukaryotes, *C*. *neoformans*, *C*. *albicans*, and *U*. *maydis* had the lowest values for cysteine composition, having in common their belonging to the fungal kingdom, the ability to become infectious and sharing pathogenicity mechanisms. *C*. *albicans* is a member of the normal human microbiome, but under certain circumstances, such as in newborns, immunodeficiencies, or multidrug therapies, it can cause mucocutaneous or systemic candidiasis. *C*. *albicans* displays a strong reaction against oxidative stress that is critical to its virulence, including for example, countering the oxidative burst response mounted by phagocytes [23]. This oxidative stress defense includes ROS detoxification systems such as H_2_O_2_ dismutation catalyzed by catalase, H_2_O_2_ detoxification mediated by peroxiredoxins and the glutathione system and reparation of oxidatively damaged protein thiols via glutaredoxins [24]. Second, *U*. *maydis* is a pathogenic fungus that causes corn smut disease and is considered a pest throughout the world, except in Mexico, where it is known as the delicacy *huitlacoche*. Interestingly, as in the case of *C*. *albicans*, the initial response of maize to *U*. *maydis* infection includes an oxidative burst elicited by membrane-bound NADPH-oxidases and cell-wall associated peroxidases, which is transient and lasts until the establishment of a biotrophic interaction [25]. Finally, *C*. *neoformans* is an opportunistic pathogen that can cause cryptococcosis (infection of the lungs and central nervous system, mainly) in immunocompromised patients. It can survive different stressors, such as hypoxia, high temperature, and oxidative and nitrosative stress. *C*. *neoformans* is phagocytosed by alveolar macrophages during the primary infection, where it has to face a strong defense mediated by reactive oxidative and nitrosative species (macrophages can contain up to 14 mM and 57 μM hydrogen peroxide and nitric oxide, respectively). To do this, this fungus has a large battery of protective systems, including the inhibition of macrophage activation, the glutathione system, and enzymes such as peroxidases, laccases, Cu Zn superoxide dismutase, and flavohemoglobin, to name a few of the most important [26]. It can be hypothesized that the low cysteine protein content of *C*. *neoformans*, *C*. *albicans*, and *U*. *maydis* is an evolutionarily selected additional defense attribute that might contribute to decreasing the oxidative stress damage to their proteins.

Finally, *G*. *lamblia* and *B*. *floridae* showed high outliers in the eukaryota group. For *G*. *lamblia*, the result is consistent with our initial empirical observation, and the possible biological reasons for this result have been mentioned in the Introduction section; mainly, the high cysteine content in the proteome of this parasite helps it cope with oxygen toxicity. *B*. *floridae* (also called Florida lancelet) is a small marine animal located in a basal position within the cephalochordata phylum and is therefore considered a fundamental model for the study of the origin of vertebrates. No studies were found in the literature that could help explain the high cysteine content in the *B*. *floridae* proteome, finding only incidental descriptions of cysteine-rich proteins in this particular organism [27, 28].

In summary, from the 7 organisms with atypical cysteine contents, 5 correspond to pathogens (*C*. *trachomatis*, *G*. *lamblia*, *U*. *maydis*, *C*. *neoformans*, *C*. *albicans*); 1 to a polyextremophile (*D*. *radiodurans*); and 1 to a marine animal (*B*. *floridae*). Additionally, organisms with unusually low cysteine contents (*D*. *radiodurans*, *U*. *maydis*, *C*. *neoformans* and *C*. *albicans*) face extreme oxidative stress conditions; whereas for *C*. *trachomatis* and *G*. *lamblia*, their particular structural and functional properties seems to be related to high cysteine content. No explanation about the significance of the results on *B*. *floridae* was found.

Nucleotide-level selection, amino acid-level selection, or a combination of both can explain amino acid composition as a major adaptive evolution feature driven by the pressure to thrive according to environmental conditions [29]. Since the two codons for cysteine (UGU and UGC) contain G and C, it must be considered whether the GC content in the genome of each species alters the probability of cysteine occurrence. In this context, it has been previously shown that the effect of correcting cysteine levels by GC content is negligible [18]. In contrast, the data from this work point toward selection at the amino acid level, since atypical cysteine contents could be related in almost all cases to specific environmental conditions, mainly oxidative stress. Furthermore, the cysteine content, even in the highest outliers, *G*. *lamblia* (2.49%) and *B*. *floridae* (2.5%), was below the expected content based on codon usage (3.28%), strongly suggesting that this amino acid has been selected against in all life domains.

An open question is how cysteine content may differ between organisms; although the data from this work cannot provide a precise answer, some hypotheses can be raised. Cysteine content is independent of proteome size, indicating that proteome expansion or contraction does not explain the change in cysteine occurrence. For low-cysteine organisms, it appears that the decrease in cysteine content could be a result of the overall decrease in the number of cysteines per protein (Table 1). Regarding proteomes with high cysteine content, these appear to be the result of the combination of the presence of specialized cysteine-rich proteins (VSP and COMC proteins) and a general increase in the number of cysteines per protein (Table 1).

## Conclusion

The data shown in this work indicate that the cysteine content in proteomes is not homogeneous across the domains of life, and it is plausible that this could be true for other amino acids. A complete and exhaustive analysis of the amino acid composition in the 3 domains of life is underway. This study, together with others in the same vein, could help to better describe the amino acid composition of proteins in living beings. Finally, we suggest starting to consider whether the typical tables for amino acid composition should be separated by domains of life instead of being shown as a whole, as is usually presented in reference works.

## Supporting information

S1 Datasheet Full list of organisms, proteome identifiers and abbreviations. (XLSX)

S2 Datasheet Cysteine content for each organism and values of the statistical analysis. (XLSX)

S1 Fig Correlation between proteome size and cysteine content. (PDF)
