# Supplementary material for: Comparison of cysteine content in whole proteomes across the three domains of life
Source: PLoS One. 2023 Nov 13;18(11):e0294268. doi: 10.1371/journal.pone.0294268 (PMC10642813; doi:10.1371/journal.pone.0294268)
Supplement: S1 Fig — (PDF) [file pone.0294268.s003.pdf]

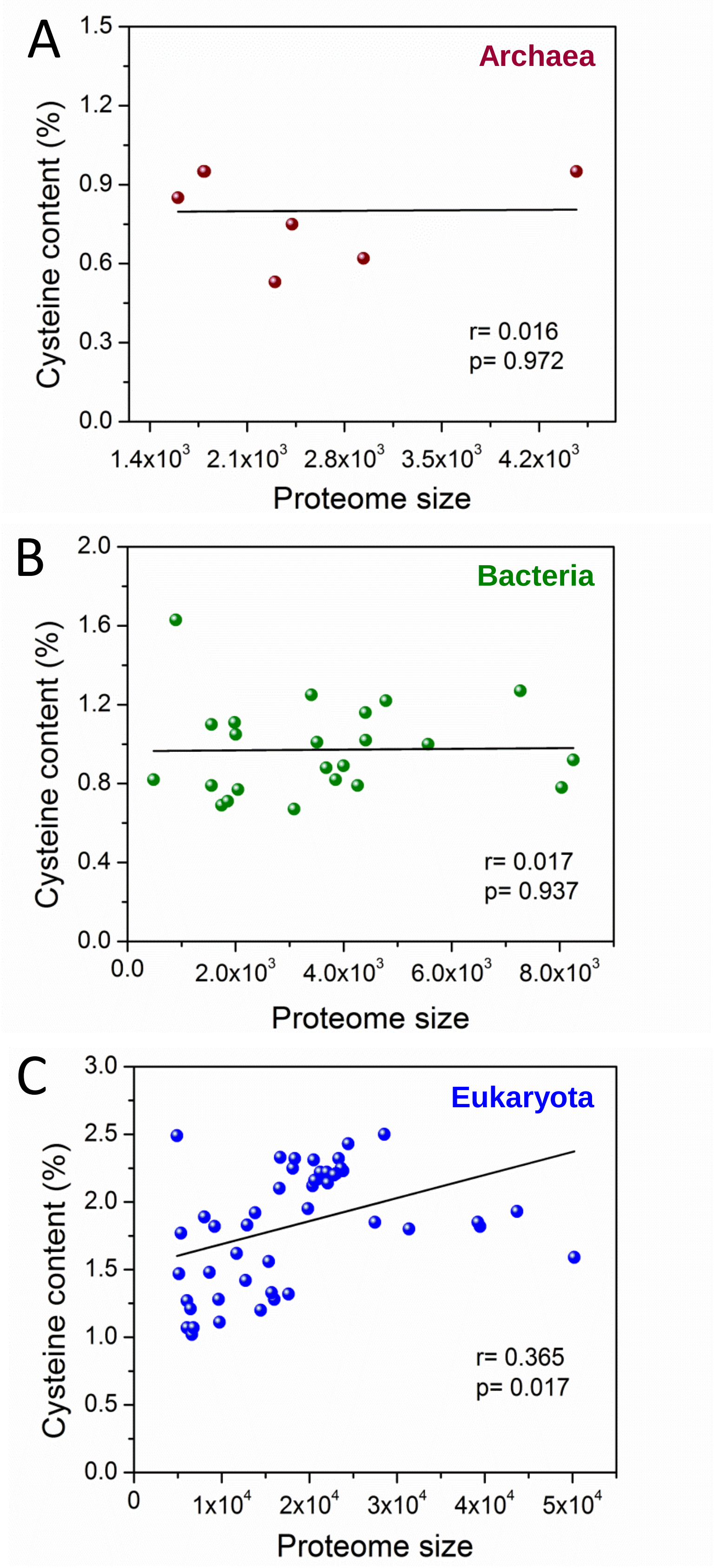

S1 Fig. Correlation between proteome size and cysteine content. The proteome size (number of proteins) versus the percentage of cysteine content was analyzed by the bivariate Pearson correlation; the lines represent the fit of the data and the insets show the Pearson correlation coefficient (r) and the statistical p-value (p).
